# Antiproliferative Effects of Ferulic, Coumaric, and Caffeic Acids in HepG2 Cells by *hTERT* Downregulation

**DOI:** 10.1155/2022/1850732

**Published:** 2022-10-28

**Authors:** Afsoon Afshari, Mahmoodreza Moein, Afshin Afsari, Zahra Sabahi

**Affiliations:** ^1^Shiraz Nephro-Urology Research Center, Shiraz University of Medical Sciences, Shiraz, Iran; ^2^Medicinal Plants Processing Research Center, Shiraz University of Medical Sciences, Shiraz, Iran; ^3^Department of Pharmacognosy, School of Pharmacy, Shiraz University of Medical Sciences, Shiraz, Iran

## Abstract

**Objective:**

Phenolic acids are well-known phytochemicals that are detected in a wide variety of medicinal plants, and their antiproliferative effects on cancer cells are known, but their mechanisms are poorly revealed. In most of cancer cells, telomerase reverse transcriptase (*hTERT*) is a dominant factor of telomere length regulation. The *hTERT* expression promotes invasiveness in tumor cells and is a hallmark of cancer. Therefore, in this study, the probable inhibitory effects of caffeic (Caf), coumaric (Cum), and ferulic acids (Fer) are investigated on the *hTERT* expression pattern in HepG2 cells.

**Methods:**

The MTT, apoptosis assays, and real-time PCR analysis were applied to evaluate viability, cytotoxicity, and *hTERT* gene expression level, respectively.

**Results:**

All of the studied phenolic acids showed cytotoxic effects on HepG2 cells in a timely manner and presented a time-dependent inhibitory effect on the growth of HepG2 cells. They reduced percentage of viable cells and induced apoptosis. Also, these phenolic acids had significant inhibitory effects on *hTERT* gene expression.

**Conclusion:**

These findings suggest that cell viability along with *hTERT* gene expression in HepG2 cells could be reduced by Cum, Caf, and Fer. As different cancer cells are resistant to conventional chemotherapeutics, this type of results proposes the telomerase as a proper target of cancer therapy development by natural products.

## 1. Introduction

Uncontrolled proliferation is one of the key features of malignant cells. Consequently, proliferation and cell death modulation might be considered as anticancer therapy [1]. Telomerase is a ribonucleoprotein complex containing reverse transcriptase activity. This enzyme synthesizes the telomere particular structure and TTAGGG repeats to protect end-caps of eukaryotic chromosomes. Also, these structures guaranty the genomic stability by preventing the degradation or fusion of chromosome ends. At the end of each cell division, telomere length shortens since DNA polymerase is unable to replicate the ends of double-stranded DNA. The reduction of this length would eventually trigger replicative senescence or aging. In normal somatic cells, telomerase is commonly inactive; therefore, telomerase activity is associated with increased proliferative capacity of normal cells and cell immortality [2].

Elevated levels of telomerase expression and/or its increased activity have been detected in the majority of human cancers [3], which can be highly related to tumor size, tumor aggressiveness, genomic instability, and prognosis of cancers [4].

According to a previous research, inhibition of telomerase activity could increase sensitivity to anticancer drugs such as etoposide and doxorubicin and radiation therapy in human breast cancer cells. So, it was suggested that controlling the telomerase levels and/or activity may be important in chemotherapy in addition to related anticancer medicines which trigger their antiproliferative responses in human cancer cells [4].

One of the most critical challenges of chemotherapy is sensitivity reduction and anticancer drug resistance in cancer cells. It might be related to translocation of telomerase subunit to mitochondria or translocation of another telomerase-associated factor (telomerase-associated protein 1, TEP1) to vaults [5]. Consequently, inhibition of telomerase activity is able to increase sensitivity of cancer to drugs like doxorubicin [5].

Based on previous research studies, natural compounds have been known to be rich sources of highly selective anticancer compounds and show promising therapeutic outcomes with few side effects [2]. Nevertheless, limited knowledge exists about the effects of natural products on telomerase activity or expression in cancer cells. In a study, the interactions of four flavonoids (luteolin, quercetin, rutin, and genistein) with the human telomeric Tel7 G-quadruplex structure were confirmed and showed their potent role in anticancer therapy by regulating the telomeric G-quadruplex structure [6].

Luteolin is a flavonoid that was able to suppress human telomerase reverse transcriptase (*hTERT*) expression in breast cancer [7]. Moreover, blackberry juice and berry-polyphenolic compounds (resveratrol and gallic acid) significantly inhibited telomerase activity in HepG2 liver cancer cells and normal human blood mononuclear cells [8].

In other study, the inhibitory effects of silibinin and curcumin on *hTERT* gene expression in a time-and dose-dependent manner in breast cancer cells (T47D) were reported [9]. The findings of another research showed that treatment with diosgenin causes downregulation of *hTERT* expression by inhibiting telomerase activity in the A549 lung cancer cell line [10]. Also, the results of studies confirmed that epigallocatechin gallate (EGCG, major polyphenol of tea) both *in vitro* and *in vivo* [11, 12] and cucurbitacin B which was extracted from Thai herb *Trichosanthes cucumerina* L. [13] showed anticancer effects by inhibiting telomerase via downregulating the *hTERT* expression.

In the present study, three phenolic acids that have different numbers of OH ring (coumaric acid (Cum), ferulic acid (Fer), and caffeic acid (Caf)) were selected. Cytotoxicity, apoptosis, and downregulation of *hTERT* gene expression were analyzed. The results were compared to know the possible relation between cytotoxic effects and their different structures.

## 2. Material and Methods

### 2.1. Cell Culture

The human cancer cell lines HepG2 were obtained from the Pasteur Institute (Tehran, Iran). They were maintained at 37°C in an incubator under 5% CO_2_ and cultured in RPMI 1640 supplemented with 10% (v/v) fetal bovine serum (FBS), 100 U/mL penicillin, and 100 mg of streptomycin/ml. After 80% confluency, cells were harvested by trypsin.

### 2.2. The Effects of Phenolic Acids on Viability of HepG2 Cells

Different concentrations (25-800 *μ*g/ml) of phenolic acids were added to the cells, and cell viability was analyzed by using modified MTT assay.

### 2.3. MTT Assay

MTT assay is performed according to modification which is made in the technique that is described previously (1983) by Mosmann. The assay is based on the ability of alive and metabolically active cells in cleaving tetrazolium salt (MTT) that results in a water insoluble substance called formazan dye. This produced dye in MTT assay dissolves in dimethyl sulphoxide (DMSO).

Briefly, HepG2 cells (10^4^ cells/well) were seeded in 96-well cell culture plates in 100 *µ*l culture medium (RPMI 1640) in 37◦C and 5% CO_2_ incubator. After 24 h incubation, the morphology of cells was seen under microscope and the medium was replaced with growth medium containing different concentrations of samples and then incubated for 24 and 48 h, separately. The medium was replaced with 10 *µ*l of MTT solutions (5 mg/ml in PBS) in 90 *µ*l of growth media and incubated at 37°C for 4h. Then, the supernatants of each well were removed and 100 *µ*L DMSO was added to each well. The plate was shaked for 15 min in a 37◦C shaker incubator. Finally, the absorbance of two wavelengths, 570 nm and 650 nm, was recorded using an ELISA reader (Awareness, microplate reader, stat fax 3200), and the resulted values were corrected against blank wells which contained growth media alone [14].

### 2.4. Determination of Apoptosis

In the apoptosis assay, cells were harvested and resuspended in staining buffer, and the Annexin V-FITC Apoptosis Detection Kit (BioLegend, USA) was used for determining the apoptosis quality. Flow cytometry was performed using the MACSQuant10 (Miltenyi Biotec, Germany), and the resulted data were analyzed using FlowJo software.

### 2.5. Gene Expression Analysis

According to data from the cytotoxicity studies, selected concentrations (600 and 1200 *µ*g/ml) of phenolic acids were used to examine gene expression. HepG2 cells were treated with different mentioned concentrations for 48 h. After incubation, cells were harvested for RNA isolation.

### 2.6. RNA Isolation and cDNA Synthesis

Total RNA was extracted by kit (Pars Azmoon) according to protocol, and the purity and concentration of extracted RNAs were calculated based on OD260/280 ratio. The cDNA was synthesized by using the Prime Script RT Reagent Kit (Takara, Japan) according to the manufacturer's protocol.

### 2.7. SYBR Green Real-Time PCR

SYBR Green Real-Time PCR method was performed in order to study the expression level of *hTERT* gene in control group and treated samples (Takara, Japan). Based on minor fluctuations, B-actin gene was selected as internal control for SYBR Green Real-Time PCR. The primers used in the reaction were designed using NCBI primer design software (ncbi.nlm.nih.gov) (Table 1).

The program used for real-time PCR reaction was as follows: initial denaturation at 95°C for 2 min, followed by 40 cycles of denaturation at 95°C for 15 seconds, annealing at 60°C for 20 seconds, and extension at 72°C for 30 seconds. Finally, amplicons were assessed by melting curve analysis.

### 2.8. Statistical Analysis

Expression levels of *TERT* following treatment are normalized to control untreated cells, calculated using the ΔΔC_T_ method. The difference in mRNA levels of *hTERT* between control and treated cells was evaluated by ANOVA and Tukey's test. Statistical analyses were performed using SPSS software (SPSS : An IBM Company, version 24.0, IBM Corporation, Armonk, NY, USA), and *p-*values less than 0.05 were considered significant.

## 3. Results

### 3.1. Cell Viability Measurement

The effects of phenolic acids on the HepG2 cell growth and proliferation were investigated by exposing them to increasing doses of Fer, Cum, and Caf for 24 h, 48 h, and 72 h. The cell viability was determined by the MTT assay, and the results of concentrations of the compounds which reduced proliferation of cells 50% (IC_50_) after 24 h, 48 h, and 72 h incubation times are shown in Table 2.

### 3.2. Apoptosis Analysis

**Figure 1:**
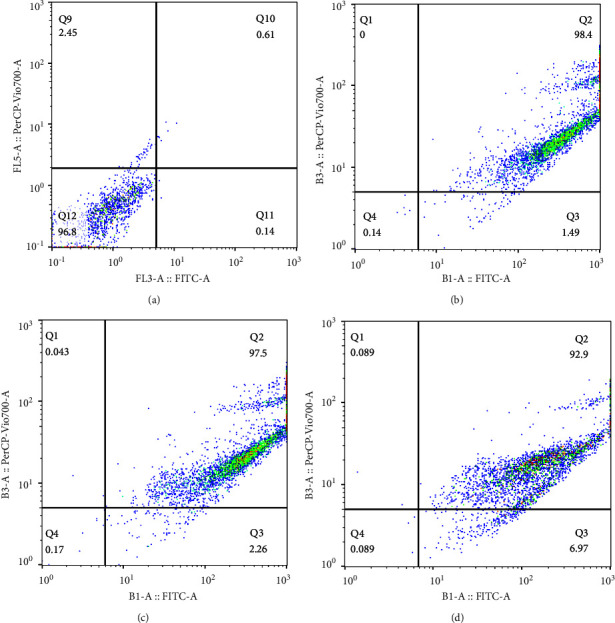
Effect of treatment of phenolic acids on HepG2 cells viability. (a) Negative control, cell (b) Ca, (c) Fer, and (d) Cum. Annexin V and 7-AAD staining was represented by flow cytometry, panel: *Q*1 = necrotic; Q2: late apoptotic cells; Q3: early stage apoptotic cells; Q4: normal viable cells.

The effects of phenolic acids on cell apoptosis were detected by using flow cytometric analysis (Figure 1 A, B, C, and D). The results indicated that the cell viability is 96.8% in negative control. The cell viability percentages were reduced to 0.13, 0.81, and 0.66% after treatment in comparison to the control group (Table 3). While apoptotic percentage was 0.75 in control, this percentage was more than 95% in other cells treated with Cum, Caf, and Fer.

### 3.3. *hTERT* Expression Level

**Figure 2:**
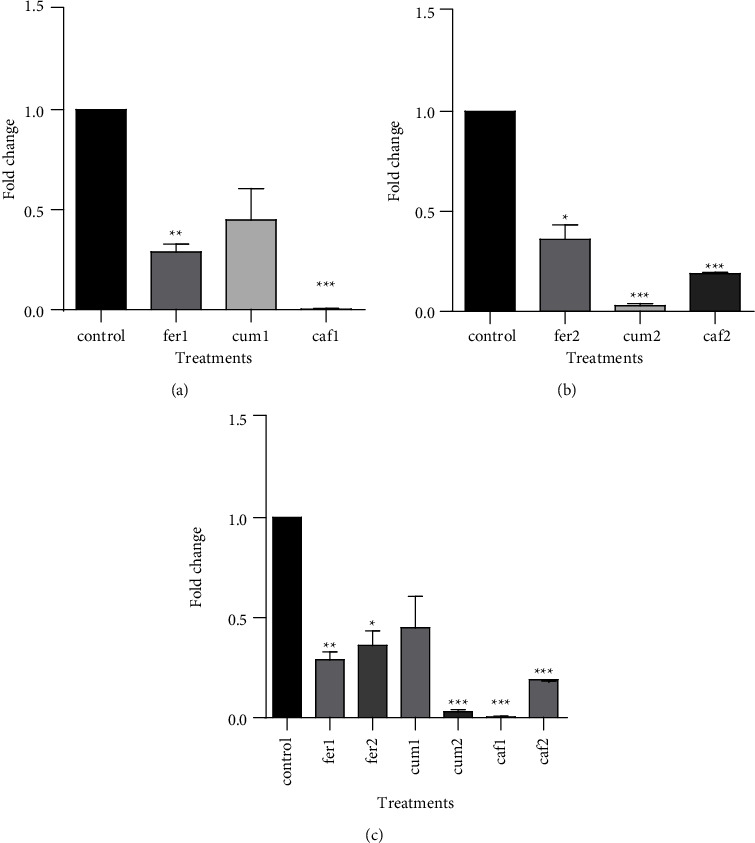
Transcription level of *hTERT* using real-time PCR experiments. The mean ± SD values of three repeats of each sample are presented. (a) Cells treated with the concentration equal to 600 *µ*g/ml. (b) Cells treated with the concentration equal to 1200 *µ*g/ml. Fer : ferulic acid, Cum : coumaric acid, and Caf : caffeic acid. All values are expressed as mean ± SD.; *n* = 3 per group; *p* < 0.05, ^*∗*^*p* < 0.001^*∗∗*^*p* < 0.001^∗∗^, *p* < 0.001^*∗∗∗*^vs control group.

The *hTERT* expression levels were evaluated through determining the ability of Cum, Fer, and Caf to change this enzyme expression in the HepG2 cells. According to our results, after normalization against *β*-actin, the levels of *hTERT* decreased significantly in groups treated with 600 *µ*g/ml Fer and Caf in comparison with the control group (*P* < 0.005 and *P* < 0.001) (Figure 1(a)). Treatment of cells with 1200 *µ*g/ml showed significant decrease in *hTERT* expression levels when compared with nontreated subjects. The *P-*value is < 0.5 and <0.001 for Fer and *P* < 0.001 for Caf and Cum (Figure 2(b)). Increased treatment dose to 1200 *µ*g/ml in Caf and Fer cases reduced *hTERT* expression levels in comparison to cells treated with 600 *µ*g/ml (Figure 2(c)).

## 4. Discussion

Eukaryotic telomerase comprises a catalytic protein subunit recognized as telomerase reverse transcriptase component (*hTERT*). Expression of telomerase is low in the normal cells; nevertheless, its expression is approximately 85-90% in tumor cells [15, 16]. Telomerase plays a critical role in the lifespan, cell development, aging, and tumorigenesis. Therefore, the modification of telomerase activity could be a potential therapeutic strategy for the treatment of human cancers [16, 17].

It seems that most of cancer cells have short telomeres and express high levels of telomerase in comparison to normal cells [18, 19]. Telomerase inhibition is a proper strategy to reduce telomeric length during cell cycles and induces growth arrest and cell death [13]. As natural products show few side effects than synthetic compounds, potential anticancer effects of natural products could be more considered in therapeutic strategy [15].

Induction of apoptosis pathways is considered as a significant method to predict the effectiveness of antitumor compounds [18]. The results of Kampa et al. study indicated that phenolic acids inhibit cancer cell growth. In this assay, among different phenolic acids, caffeic acid was the most potent inhibitor of cell growth. Potential inhibitory effects of these phenolic acids on T47D human breast cancer cells growth were followed by this order: caffeic acid > ferulic acid = protocatechuic acid = PAA > sinapic acid = syringic acid. The suggested mechanisms were the direct interaction with the aryl hydrocarbon receptor and inhibitory effects on nitric oxide synthase [20].

To evaluate the effects of phenolic acids on cell proliferation, we measured their effects on the growth of HepG2 cells. These compounds markedly decreased cellular proliferation in a time-dependent manner. Flow cytometry analysis showed that apoptotic cells increased after the cells were treated with concentrations (IC_50_) of phenolic acids for 48 h. The significant differences were observed in the proportion of Annexin V-positive cells in treated and control cell groups.

A study revealed that caffeic acid induces apoptosis by the mitochondrial pathway. This phenolic acid induced the release of cytochrome C, downregulation of Bcl-2, and upregulation of p53 protein expression [21]. Also, Chung et al. reported that the caffeic acid and caffeic acid phenethyl ester inhibited hepatoma growth through inhibition of NF-B as well as MMP-9 catalytic activity in HepG2 cells [22].

According to previous reports, presence of OH ring substituents in phenolic acids structure is a determinative factor in their effects on cancer cells. Other factors comprise length of the carbon chain between the aromatic ring and the terminal carboxylic and the presence of a double bond in the carbon chain [23]. Phenolic acids have been detected to have anticancer effects on different cells types [24–27]. Therefore, in the current study, it is tried to investigate the influence of these three phenolic acids on the expression of the telomerase reverse transcriptase.

The results of this study determined the most inhibitory effects on *hTERT* gene expression are related to Caf > Fer > Cum, respectively. During the increase of concentration, this trend changed to Cum > Caf > Fer.

Based on previous reports, the number of OH ring substituents was known as the main factor in anticancer effects of those phenolic acids, and it seems that triphenol phenolic acid was more toxic than diphenols in different investigated cancer cells. Caf is a dihydroxylated phenol while Fer and Cum have one OH in their structure. It seems that these results could be described by the number of hydroxyl groups in these phenolic acids [23].

According to our results, increase in the concentration of Caf and Fer leads to elimination in the effects of these phenolic acids which resulted in the downregulation of *hTERT* gene expression. Based on previous studies, elevated concentration of phenolic compounds plays an important role in pro-oxidants in cells [28]. While, low concentrations of phenolic acid scavenged free radicals and play a role as an antioxidant [29–32]. Also, Du et al. reported that the order of pro-oxidant activity of phenolic acid was as follows: chlorogenic acid > ferulic acid > caffeic acid > salvianolic acid B [29]. On the other hands, telomeres are prone to oxidative damage, and oxidative stress is able to disrupt telomere maintenance. Therefore, exposing cells to ROS leads to an increase in telomerase expression [33]. Consequently, in this study, high concentrations of Fer, Cum, and Caf might play roles of pro-oxidant in HepG2 and are unable to reduce *hTERT* gene expression in comparison to lower concentration since in oxidative stress condition telomerase expression was increased.

In a previous study, 3 *μ*M of caffeic acid undecyl ester (CAUE) induced a concentration-dependent decrease in *hTERT* expression and produced complete inhibition of telomerase activity and *hTERT* expression [34]. In another study, Fer improved the bioavailability of *δ*-tocotrienol (T3), hence increased the inhibitory effects of *δ*-T3 on the telomerase [35].

According to previous results, activation of oncogene c-MYC is a modulatory factor in *hTERT* expression. Also, the *hTERT* promoter has a number of c-MYC binding sites. Consequently, the c-MYC binding region of the *hTERT* might be the direct target of natural compounds [36]. For instance, gambogic acid (GA), isolated from the gamboge resin of *Garcinia hanburyi* tree [36], wogonin, an active compound in *Scutellaria baicalensis* [37], and curcumin [39] and ginger (*Zingiber officinale* Roscoe) extracts [39] reduced the expression of c-MYC. Accordingly, it would be suggested that phenolic acids might have another anticancer property through the inhibition of c-MYC transcription [40].

Ramachandran et al. reported that the MCF-7 cells were treated with curcumin, and this treatment decreased telomerase activity and *hTERT* mRNA expression. Also, curcumin was able to prevent telomerase activity and *hTERT* expression in brain tumor cells [40].

Our study showed that anticancer activity of phenolic acids can be mediated by reducing proliferation as well as downregulation of *hTERT*. It seems that caffeic, coumaric, and ferulic acids would be promising natural products in cancer treatment. Further studies are necessary to explore the effects of these compounds on activity of telomerase, which is the limitation of our study.

## 5. Conclusion

Antiproliferative effects of phenolic acids in HepG2 cells are mediated by *hTERT* downregulation. Inhibition of telomerase is one of the attractive approaches for hepatic cancer treatment. Natural products recommend different potent compounds. Phenolic acids are a class of natural compounds, and are promising candidates in cancer treatment which might be used in the design of future medicines.

## Figures and Tables

**Table 1:** Sequences of forward and reverse primers.

Studied gene	Primer sequence
HTERT	
**Forward (5′-3′):** **Reverse (5′-3′):**	CTGACGTGGAAGATGAGCGT CTCATCAGCCAGTGCAGGAA
Human *β*-actin **Forward (5′-3′):** **Reverse (5′-3′):**	TGGCACCCAGCACAATGAA CTAAGTCATAGTCCGCCTAGAAGCA

**Table 2:** IC_50_ results of phenolic acids in different incubation times in HepG2 cells.

Sample	IC_50_ (*µ*g/ml) 24 h	IC_50_ (*µ*g/ml) 48 h	IC_50_ (*µ*g/ml) 72 h
**Cum**	846.66 ± 45.88	798 ± 70.31	511.11 ± 28.34
**Fer**	869.16 ± 14.21	782 ± 91.79	736.66 ± 13.61
**Caf**	916.66 ± 80.36	627.45 ± 202	608.33 ± 70.94

**Table 3:** Effects of phenolic acid treatment on cell viability.

Cell number counted (%) Sample	Viable	Apoptotic	Necrotic
**Negative control**	96.84 ± 0.74	0.84 ± 0.085	2.1 ± 0.49
**Caf**	0.13 ± 0.016	98.76 ± 0.75^*∗∗∗*^	0.14 ± 0.02
**Fer**	0.81 ± 0.05	99.53 ± 0.15^*∗∗∗*^	0.7 ± 0.1
**Cum**	0.66 ± 0.04	99.75 ± 0.05^*∗∗∗*^	0.45 ± 0.06

Data are expressed as the means ± SD of triplicate in independent experiments. Significant difference considered *P* < 0.001 (^∗∗∗^: compared to the negative control group).

## Data Availability

The data used to support the findings of this study are included within the article.
